# Clinical Toxoplasmosis in Dogs and Cats: An Update

**DOI:** 10.3389/fvets.2019.00054

**Published:** 2019-02-26

**Authors:** Rafael Calero-Bernal, Solange M. Gennari

**Affiliations:** ^1^Saluvet Group, Department of Animal Health, Complutense University of Madrid, Madrid, Spain; ^2^Program of Master's in Animal Medicine and Welfare, Faculty of Veterinary Medicine, University of Santo Amaro, São Paulo, Brazil; ^3^Faculty of Veterinary Medicine, University of São Paulo, São Paulo, Brazil

**Keywords:** *Toxoplasma gondii*, dogs, cats, clinical disease, symptoms, genotype, differential diagnosis

## Abstract

Toxoplasmosis is caused by the globally distributed protozoan parasite *Toxoplasma gondii* (phylum Apicomplexa); the disease can be clinically important for almost all homeothermic animals, including birds and humans. Toxoplasmosis course involves general clinical signs, such as fever, anorexia, or dyspnea, and more specific signs with neural, respiratory, cutaneous, or ocular involvement. Because of the wide range of clinical signs, the diagnosis in domestic and pet animals can be complicated. Hence, this review aims to provide a comprehensive analysis of some scarcely discussed aspects of toxoplasmosis, such as ocular and cutaneous manifestations, congenital infections, influence of *T. gondii* genotype on clinical toxoplasmosis, and recent findings regarding differential diagnosis. This review could be of special interest to clinicians and researchers.

## Introduction

Toxoplasmosis is caused by the globally distributed intracellular protozoan parasite *Toxoplasma gondii* (phylum Apicomplexa, family Sarcocystidae). The disease has a complex epidemiology; the parasite is capable of infecting virtually all warm-blooded animals, and has a two-host life cycle (1). Domestic cats and other felids are the definitive hosts (DHs). All non-feline animals, including dogs and humans, are intermediate hosts (IHs), however, *T. gondii* can also undergo asexual reproduction in Felidae that act as IHs. There are three stages in the protozoan's life cycle that explain its biological success. First, tachyzoites multiply actively in tissues, quickly spread to almost all organs, and cause most of the pathology. Once they reach specific tissues (central nervous system, muscle, and viscera), they convert into bradyzoites which remain latent in a cyst form leading to a life-long chronic infection until a DH ingests the tissue. Then, bradyzoites are released and penetrate the small intestine epithelial cells, giving rise to schizonts that form gamonts and, finally, oocysts. Oocysts constitute the environmentally resistant (and infective) stage. Hosts can become infected horizontally by ingesting tissues containing cysts, consuming water or food contaminated with oocysts, or by transfusion or transplantation with parasitized organs. They can also result infected vertically, by congenital infection.

In general, *T. gondii* infection is associated with a low rate of morbidity and mortality in dogs and cats, but clinical consequences for primary care clinics should be taken into account. Clinical toxoplasmosis is more frequent in cats than in dogs, which commonly suffer from neosporosis by *Neospora caninum*, and among them, non-vaccinated animals are more susceptible.

On review of the available literature (www.ncbi.nlm.nih.gov), ~79 clinical cases in cats and 45 in dogs have been comprehensively reported after 1989, when *N. caninum* was newly described causing toxoplasmosis-like disease in dogs (1). Numerous studies have shown seroprevalence and/or parasite prevalence ranges in dogs and cats from 6 to 88% worldwide [reviewed in reference (1)]. Seroprevalence status in cats is essential regarding zoonosis prevention and has been recently reviewed elsewhere (2).

Other review papers have focused on toxoplasmosis diagnosis and treatment. In the present article, the authors aimed to highlight less known clinical manifestations, to review aspects regarding differential diagnosis, and to determine how strain/genotype may be involved in the clinical presentation of the disease, which could be of special interest for clinicians and researchers.

## Clinical Presentation and Pathology of Toxoplasmosis in Dogs and Cats

Clinical cases of toxoplasmosis are much more frequent in cats than in dogs (1, 3), which mostly suffer from neosporosis (4). A high proportion of *T. gondii* clinical infections are triggered by immunosuppressive chemotherapy (5). Cases of co-infections that can aggravate a process by *T. gondii* (6, 7) are discussed in the respective section of the present paper.

Dogs rarely suffer from toxoplasmosis as a primary disease, and, in most cases, the disease is linked to immunosuppression and absence of vaccination against canine distemper virus (CDV). Neurological disease, with signs of seizures, cranial nerve deficits, tremors, ataxia, and paresis or paralysis within encephalomyelitis (8), may be seen. Paraparesis and tetraparesis that progressed to lower motor neuron paralysis and nodules in the spinal cord, were described in a dog with dual infection with *Sarcocystis neurona* and *T. gondii* (6). Other reported cases of toxoplasmosis in dogs include: noise sensitivity in an 8-year-old female collie (9); myositis that initially showed an abnormal gait, muscle wasting, and stiffness (10); and ocular disease described as necrotizing conjunctivitis (11), anterior uveitis, endophthalmitis, and chorioretinitis (12).

Cutaneous manifestations are generally associated with immunosuppression after corticoid therapy and transplantation (13–17). Lesions are characterized by erythematous epidermal nodules, pyogranulomatous and necrotizing dermatitis, and panniculitis, with multifocal vasculitis and vascular thrombosis. Zoites are frequently found in lesions. In general, clindamycin is the treatment of choice for cutaneous toxoplasmosis (14), although *N. caninum* is the most frequent coccidian causing skin lesions in dogs, what should be considered in a differential diagnosis (4).

In cats, clinical toxoplasmosis is more severe in transplacentally infected kittens (18), which frequently develop hepatitis or cholangiohepatitis, pneumonia, and encephalitis and show signs of ascites, lethargy, and dyspnea. In adults, unspecific clinical signs can be observed (19, 20). The occurrence of hepatitis and abdominal involvement, hepatic failure, and hyperplastic cholangitis has been described yet (21, 22). In addition, extra-intestinal enteritis (21), inflammatory intestinal disease (23), thickening of the gastric wall due to eosinophilic fibrosing gastritis, and regional lymphadenopathy (21, 24) were noted. The disease may be rapidly fatal in cats with severe respiratory or neurological signs (18).

Pneumonia is the main sign of generalized toxoplasmosis (25), and acute respiratory distress syndrome and septic shock may occur (26). Ocular toxoplasmosis has been observed in cats without poly-systemic clinical signs of disease, and anterior or posterior uveitis, iritis, iridocyclitis, or chorioretinitis. Aqueous flare, keratic precipitate, lens luxation, glaucoma, and retinal detachment are common manifestations of uveitis (19). Therefore, ocular fundic examination should be a routine part of the examination in febrile cats (1). Less frequent findings, such as myocarditis with echocardiographic changes (27), diarrhea with oocysts (28), or pyogranulomatous cystitis after renal transplantation (29), were also reported. Primary or reactive neurological disease is frequent in cats; infection of the encephalon (30), spinal cord, and nerves has been identified (31–33). In general, myelitis, marked generalized mononuclear cell inflammation of the gray matter, non-suppurative encephalitis, and perivascular cuffing are common findings. Cases of intracranial granuloma (34, 35) and panencephalitis (36) have been also addressed.

Generalized toxoplasmosis in immunocompetent and immunosuppressed cats, involving acute interstitial pneumonia, acute and multifocal necrotizing hepatitis, non-suppurative meningoencephalitis with glial granuloma, moderate lymphadenomegaly, and splenomegaly, have been reported (37–40). In addition, cutaneous manifestations with nodules that may ulcerate are sometimes related to feline immunosuppression (40–42).

## Congenital Infections in Dogs and Cats

Most of infections in dogs and cats occur horizontally, but infectivity by the oral route depends on the parasite stage ingested, this issue has been revised thoroughly elsewhere (1). By contrast, the aspect of congenital infections in dogs and cats has not been reviewed in depth in the literature. After primo-infection during pregnancy, parasitemia can cause placentitis, followed by spread of tachyzoites to the fetus. Little is known about transplacental toxoplasmosis in dogs, although its occurrence is thought to be less common than in other species (1). The only evidence is based on two studies (43, 44) that reported a high prevalence of *T. gondii* DNA detection in fetal tissues of dogs, but the significance of these findings is unknown.

In dogs, *T. gondii* was isolated from pups from a seropositive bitch in Australia; however, no clinical signs were seen in any of the animals (45) (Table 1). *T. gondii* DNA was detected in fresh semen from five out of eleven seropositive naturally infected healthy dogs in Brazil, and *in vitro* isolation of the parasite was achieved (50). However, venereal transmission had been demonstrated earlier, when fresh semen, collected from experimentally infected dogs that tested positive for *T. gondii*, was used for artificial insemination (AI) of four naïve bitches. Seroconversion was observed in all females seven days after AI, and reabsorption occurred in two of the dogs. The remaining bitches sustained full-term gestations, and *T. gondii* cysts were detected in brains of four offspring (51).

**Table 1:** Summary of the clinical cases in dogs in which *T. gondii* strains were genotyped.

**Clinical presentation**	**Patient**	**Country, year of diagnosis**	**Parasite isolation (isolate designation)**	**ToxoDB RFLP genotype number**	**History**	**References**
Congenital toxoplasmosis (Concurrent infection with viral pathogens).	43-day-old mixed-breed puppy, showing convulsions, blindness, and spontaneous death. Seroreactivity to *T. gondii* of the dam is associated with the systemic disease observed in the puppy.	Brazil, 2013	No	Type III (SAG3): ToxoDB incomplete allele profile.	Concomitant infections of CDV, CAdV-1, CAdV-2, CPV-2 and *T. gondii*; other 14 siblings of this puppy, born to a 10-month old dam, which was seropositive (titer: 1,024) for *T. gondii*, also died. Necropsy revealed unilateral corneal edema (blue eye), depletion of intestinal lymphoid tissue, non-collapsible lungs, congestion of meningeal vessels, and a pale area in the myocardium.	(7)
Neuromuscular disease	A 6-month-old female stray dog with muscular atrophy of the femoral region and hyperextension of hind limbs. Poor body condition.	Italy, 2016	No	Type I (RFLP-SAG2 and 12 MS markers).	Serologically positive to *T. gondii* IgG antibodies and negative to *N. caninum*. PCR of muscle biopsy resulted positive to *T. gondii*. The dog recovered after 4 weeks of treatment with clindamycin hydrochloride and aquatic physiotherapy.	(10)
Cutaneous toxoplasmosis	A 2-year-old male mixed-breed dog, found on the street and adopted. Two month later, the dog was diagnosed with severe erythroid and myeloid aplasia, megakaryocytic aplasia, myelonecrosis with lymphoplasmocytic infiltration, and grade II fibrosis.	Brazil, 2014	Yes, in mice, from skin aspirate. TgDgBr20 characterized as mouse virulent.	Type BrI (ToxoDB #6) using PCR- RFLP and as Africa 1 through microsatellite analysis.	After initial diagnosis, immunosuppressive therapy was initiated with prednisone. Two and a half months later, the dog presented dermal lesions, initially small, hard, and slightly erythematous that rapidly evolved to large hard nodules that ulcerated with drainage of purulent material. Structures compatible with tachyzoites were found in the skin aspirate. IFAT *T. gondii* antibodies were detected, with a titer >65,536.	(16)
Congenital infection (no clinical signs in mother nor pups)	18-month-old female cattle dog that gave birth to 6 seronegative pups	Australia, 2007	Yes, in mice from brains of pups (TgDgAu1); also bioassay in cats.	Type II (10 RFLP markers): ToxoDB#1	Bitch had *N. caninum* IFAT >1:2,800. Bitch and pups negative to *T. gondii* (LAT). Bitch positive for both by WB. By PCR, all 6 pups positive for *T. gondii* and 2 pups also for *N. caninum*.	(45)
Neurological signs	111 dogs admitted in veterinary services with neurological symptoms: ataxia, seizures, behavioral changes, paralysis and paraplegia of members, and tremors.	Brazil, 2001–2002	Yes, in mice, from brain (no ID provided)	9 isolates (SAG2 marker): 4 of type I, and 5 of type III (ToxoDB incomplete allele profile)	34 out of 111 dogs euthanized because of the severity of neurological signs. Eleven of the 34 euthanized dogs were *T. gondii* IFAT positive (IFAT > 1:16). The 9 isolates were from seropositive dogs.	(46)
Neurological signs	A 3-year-old female cocker spaniel presented a mucopurulent ocular discharge, bloody diarrhea, polyuria, lymphadenopathy and neurological signs including circling, tetraparesis, and left hindlimb hyperextension.	Brazil, 2006	Yes, in mice, from brain. *T. gondii* antibodies (IFAT = 1,024) were observed before euthanize.	Type I (SAG2 marker)	Toxoplasmosis, erhlichiosis and distemper virus co-infection in a dog with an exuberant neuropathological clinical picture.	(47)
Neurological signs	50 dogs admitted in veterinary services with neurological symptoms: ataxia, seizures, behavioral changes, paralysis and paraplegia of limbs, and tremors.	Brazil, 2009–2010	Yes, in mice, from brain of 7 of 11 seropositive dogs. TgDogBr (btu1-3)	11 markers, showed: 1 unique genotype (btu1), and 2 known genotypes TgCatBr1 (ToxoDB #11), and P89 (ToxoDB #8).	11 out of 50 (22%) dogs were *T. gondii* positive (IFAT >1:16), and the parasite was isolated from 7 out of 11 positive dogs. In 2 dogs *N. caninum* was also isolated in gerbils.	(48)
Co-infection with *Leishmania braziliensis*	4-year-old female mixed breed, showing hyporexia, diarrhea with mucous, and tachypnea. Suspected bronchopneumonia, and died 10 days after the beginning of treatment.	Brazil, 2013	No, direct genotyping from lung, kidney and liver tissues.	11 markers showing profile of the isolate TgCTBr5: ToxoDB#19.	Pulmonary fields with diffuse opacification on a thoracic radiographic image with bronchial, interstitial, and alveolar patterns, and enlargement of the bronchial wall, suggesting bronchopneumonia.	(49)

*CDV, Canine distemper virus; CAdV-1, Canine adenovirus A type 1; CAdV-2, Canine adenovirus A type 2; CPV-2, Canine parvovirus type 2; IFAT, immunofluorescence antibody test; LAT, latex agglutination test; WB, western blotting; PCR, polymerase chain reaction; RFLP, restriction fragment length polymorphism*.

Before 2010, scientists were not aware of any confirmed report of natural congenital toxoplasmosis in dogs (1), but there was experimental evidence for it. It was confirmed when three bitches were fed 15,000 sporulated *T. gondii* oocysts at 32, 40, and 56 days of gestation, and two of the three dogs infected during pregnancy showed evidence of congenital infection, while one aborted (52). Further studies also showed transplacental transmission with miscarriage and fetal death in naturally infected bitches challenged with subcutaneous and oral administration of tachyzoites and oocysts, respectively (53).

On the other hand, kittens born to queens infected with *T. gondii* during gestation can become infected transplacentally or via suckling (54); in general, clinical illness is common and severity varies with the stage of gestation at the time of infection (1). Congenital toxoplasmosis was diagnosed histologically in nine kittens and one queen from five litters. The queen died of generalized toxoplasmosis, and the kittens presented with toxoplasmic hepatitis and pneumonia; in addition, three 1-month-old kittens from another litter were shedding *T. gondii*-like oocysts (18). After oral inoculation of five full-term pregnant queens with *T. gondii* tissue cysts, the clinical signs and lesions were comprehensively described in a pioneer study (54); twenty-two live and three dead kittens were born 16 to 31 days after inoculation. A wide range of histologic lesions included: proliferative interstitial pneumonia, necrotizing hepatitis, myocarditis, skeletal and glossal myositis, non-suppurative encephalitis affecting the cerebrum, brain stem, and spinal cord, uveitis, necrotizing adrenal adenitis, and interstitial nephritis. Also, placental lesions consisting of grossly visible areas of necrosis and mineralization were observed.

Recent articles reporting on congenital infection in cats are scarce. In Turkey, an interesting case of fatal systemic toxoplasmosis with necrotic pneumonia involving a 2.5-year-old pregnant queen and its kitten was reported (55).

Experimental infection of queens in the middle third of pregnancy, using two different Brazilian isolates (BrI, BrIII), showed almost no difference in abortion and premature stillbirth rates (56). Venereal transmission in cats does not appear to occur; *T. gondii* was not detected in the semen, testicles, or epididymis tissues of primo-infected cats, after challenging with *T. gondii* tissue cysts and tachyzoites (57). Infection through milk ingestion in cats is supported by results, such as the detection of *T. gondii* by bioassay and PCR in milk of nursing queens after experimental infection (58). Cats of any age may die or develop severe disease following parenteral inoculation with tachyzoites, bradyzoites, or oocysts, and large doses of corticosteroids can aggravate clinical toxoplasmosis (1). Cats infected during pregnancy can develop placentitis, and congenitally infected kittens, severe toxoplasmosis, including ophthalmitis (54, 59–61).

## Immunosupression and Clinical Toxoplasmosis

Vast literature has been published since initial studies (1, 3, 62) exposed that viral infections in dogs and cats may predispose to clinical toxoplasmosis; however, the results are not conclusive. A few cases of feline clinical toxoplasmosis combined with feline immunodeficiency virus (FIV) (63) and feline leukemia virus (FeLV) (40) have been described. During experimental infections, challenge with FIV triggered the disease and predisposed cats to acute generalized toxoplasmosis (64), but later studies on the general population did not identify the same association (65).

Organ transplantation has been identified as a cause of acute toxoplasmosis; three cats and one dog developed signs 3–6 weeks after renal transplantation (13). Furthermore, pyogranulomatous cystitis was observed in a cat 6 weeks after the same surgery (29). All animals had received immunosuppressive therapy. Therefore, consideration should be given to determining the serological status against *T. gondii* prior to use drugs that are potent inhibitors of cell-mediated immunity, such as cyclosporine. It has been reported in the literature that skin nodules worsened after treatment with corticosteroids in two cats (41, 66), and three cases of generalized toxoplasmosis with pneumonia in cats were observed after cyclosporine (5, 67) and prednisolone (68) treatments. In another case, disseminated toxoplasmosis involving acute respiratory distress syndrome and septic shock occurred in a cat (26) after the use of cyclosporine for eosinophilic dermatitis treatment. Several dogs developed cutaneous toxoplasmosis after treatment with prednisone and cyclosporine against immune-mediated thrombocytopenia and immune-mediated hemolytic anemia (14, 16, 17, 69). Moreover, topical anti-inflammatory therapy required to control keratoconjunctivitis sicca in a dog triggered a necrotizing conjunctivitis by *T. gondii* (11).

The re-shedding of oocysts by cats remains an important aspect in the epidemiology of *T. gondii*. It was previously believed that, after the first infection, cats would excrete thousands of oocysts, and then elimination would no longer occur during the life of the animal. However, recent studies have demonstrated re-shedding after experimental application of immunosuppressive therapy (70). In addition, it was reported that clinically ill cats can shed oocysts (28), suggesting that special precautions must be taken during clinical care. Besides, cats that were experimentally re-infected with *T. gondii* at 12, 24, and 36 months after the primary infection, re-excreted oocysts and the amount was higher the longer the time after the primary infection, especially when a heterologous strain was used (71).

## *Toxoplasma Gondii* Genotype and Clinical Toxoplasmosis

One recurrent question is how the *T. gondii* strain (genotype, http://toxodb.org/toxo/) relates to the occurrence of clinical disease. This concept is still under study. In a recent paper carried out in Australia (20), the authors compared the genotypes of *T. gondii* in latently infected cats with those in cats suffering from clinical toxoplasmosis, by direct genotyping of the DNA isolated from tissue samples. ToxoDB genotype #3 was commonly found among both sample sets, suggesting that the *T. gondii* genotype is not a determinant of clinical disease in naturally infected cats in Australia.

The current knowledge regarding *T. gondii* strain/genotype distribution in clinical cases of dogs and cats is summarized in Tables 1,2, respectively. Only ToxoDB#3 has been detected in five feline clinical cases from two different countries, Australia (20) and Switzerland (37); therefore, no specific genotype can be associated with a certain clinical outcome or presentation. Thus, whether disease depends on immunological status, infection dose, co-infection rates, and geographical location (understood as genetic variant distribution), rather than on “specific genotype” involvement, is still under debate. It can be concluded that there is no specific cluster of strains associated with clinical toxoplasmosis; hence, further studies based on isolation and strain genotyping will be of major interest, especially in locations where a wide range of *T. gondii* genotypes have been described in animals and humans.

**Table 2:** Summary of the clinical cases in cats in which *T. gondii* strains were genotyped.

**Clinical presentation**	**Patient**	**Country, year of diagnosis**	**Parasite isolation (isolate designation)**	**ToxoDB RFLP genotype number**	**History**	**References**
Generalized toxoplasmosis and latent asymptomatic cases	Tissue samples from 16 cats seropositive for *T. gondii* and from 1 cat previously diagnosed with disseminated toxoplasmosis	Australia, 2016	No	ToxoDB genotype#3 (12 markers) in 7 cats; ToxoDB#1, #3, #128, or #129 (6 markers) in 1 cat	Seven cats, including four with clinical toxoplasmosis were genotyped as ToxoDB genotype#3 using 12 loci. One cat, with latent infection could only be typed at six single copy loci; the virtual RFLP suggested genotype ToxoDB#1, #3, #128, or #129.	(20)
Generalized toxoplasmosis	A 6-month-old domestic male cat was hospitalized because of lethargy, anorexia, fever, diarrhea, and respiratory difficulty of 1-wk duration.	USA, 1997	Yes, bioassay in mice with oocysts shed in cat feces. (TgCatUs9)	*T. gondii* (ToxoDB#4) 10 PCR-RFLP markers. Genotype #4 is grouped into the Type 12 lineage that is dominant in wildlife from North America.	Six million *T. gondii* oocysts were found in one sample of cat feces. Antibodies to *T. gondii* (MAT = 1:800) were positive in its serum. The cat was medicated for 10 days (Clindamycin) and became asymptomatic.	(28)
Fatal generalized toxoplasmosis	A 10-year-old male, neutered domestic shorthair cat presented 1-week history of fever, anorexia, vomiting, and diarrhea. Animal was fully immunocompetent.	Switzerland, 2011	Yes, bioassay in mice and culture in Vero cell line.	9 PCR-RFLP markers ToxoDB#3 (PRU-type strain).	Seronegative for FIV and FeLV. Fine-needle aspirates of mesenteric lymph nodes revealed the presence of banana-shaped apicomplexan parasites. The cat died after 4 days of hospitalization.	(37)
Generalized toxoplasmosis	Among 193 cats submitted for necropsy during a 3.5-year period, 6 (3.1%) had been diagnosed with generalized toxoplasmosis	Finland, 2008–2011	Yes, by culture in Vero cell line from brain, heart, liver, lung, and lymph nodes.	The results at the 6 RFLP markers were all fully consistent with clonal type II. Such genotype was also obtained from the oocysts of a positive fecal sample.	In all cats, patent histopathological lesions of hepatitis and pneumonic lesions were evidenced. Of the 6 cats, 5 had brain lesions.	(38)
Fatal generalized toxoplasmosis and coinfection with FeLV	A 5-year-old male mixed breed, presented intense dyspnea and died 3 days later. Animal was immunosuppressed.	Brazil, 2015	No	Genotyping of DNA extracted from lungs resulted in ToxoDB#10 (archetypal type I). Microsatellite analysis showed that the strain was a variant of type I with two atypical alleles.	The histopathological examination showed severe necrotic interstitial bronchopneumonia and mild necrotic hepatitis, associated with intralesional cysts and tachyzoites of *T. gondii* (positive by IHQ). The bone marrow showed chronic myeloid leukemia and the neoplastic cells were positive by anti-FeLV IHC evaluation.	(40)
Co-infection with, *Dirofilaria immitis*, FIV and FeLV	A total of 362 blood samples were obtained from stray and pet cats. The brain, heart, and lung of seropositive animals were subjected to DNA extraction.	China, 2014–2015	No	By 11 PCR-RFLP markers, two *T. gondii* genotypes (ToxoDB#9 and ToxoDB#1) were identified.	Antigens or antibodies to *D. immitis*, FeLV, and FIV were found in 3.0, 11.3, and 9.1 % of 362 cats. 70 out of 362 (19.3 %) cats were seropositive for *T. gondii* (MAT > 1:25).	(72)

*FIV, Feline immunodeficiency virus; FeLV, Feline leukemia virus; MAT, modified agglutination test; IHQ, immunohistochemistry; PCR, polymerase chain reaction; RFLP, restriction fragment length polymorphism*.

On the top of that, there is a wide genetic diversity within the parasites isolated from asymptomatic dogs (73, 74) and cats (72, 75), corresponding to a complex epidemiology shown in different geographical areas [(76), http://toxodb.org/toxo/].

## Recent Findings Regarding Differential Diagnosis

When congenital, cutaneous, generalized (pneumonia, myositis, etc.), or neurological toxoplasmosis-like clinical signs are identified in dogs and cats, clinicians and researchers should suspect of protozoal infections, caused by Apicomplexans (4, 77, 78). As mentioned before, clinical cases are frequently associated with immunosuppressive treatments (20, 79, 80). Differential diagnosis might be influenced by the geographical location where the clinical cases occur; for example, *S. neurona* causing encephalitis and *Sarcocystis canis* causing hepatitis have been confined to the Americas (78).

Reports of clinical protozoal infections resembling toxoplasmosis have been published, including cutaneous neosporosis in a dog (81); a cutaneous infection by a *T. gondii*-like parasite in a dog and a cat (42, 82, 83); congenital infections in dogs by *S. neurona* (84) and *N. caninum* (85); generalized disease showing pneumonia in dogs by *Sarcocystis* spp. (86), *N. caninum* (85), and lungworms (87); hepatitis in dogs by *Sarcocystis* spp. (88), *S. canis* (77), *Sarcocystis caninum, Sarcocystis svanai* (89), and *N. caninum* (79); myocarditis in dogs by the West Nile Virus (90) and *N. caninum* (91); and skeletal muscle myositis in dogs by *S. caninum* and *S. svanai* (89, 92). Also, non-infectious neurological diseases showing a wide range of signs are frequent in cats (30, 93) and dogs (94, 95). In dogs, CDV is an important cause of neurological disease, but other infectious agents include eastern equine encephalitis virus (96), *S. neurona* (97), and *N. caninum* (8, 85). Co-infections of *T. gondii* with viruses, like CDV, canine adenovirus type 1 and type 2, and canine parvovirus type 2 (7, 72), along with rickettsial bacteria (47) and protozoans, like *Leishmania* spp., *N. caninum*, and *S. neurona* (6, 15, 49, 98–100), can mask the infections, so clinicians need to pay attention to all variables.

As an instance of cutaneous toxoplasmosis was described in an immunosuppressed dog. The material collected from the skin lesions was negative for fungal and bacterial cultures, and tachyzoites were found on the Giemsa-stained smears. Serum was tested for *N. caninum, T. gondii, Leishmania infantum chagasi*, and *Leishmania amazonensis* IgG antibodies, and only *T. gondii* antibodies were detected (IFAT titer = 65,536). DNA was extracted from the collected material and examined by nested PCR, confirming the diagnosis of *T. gondii* (16) (Table 1).

## Conclusion

Clinical toxoplasmosis in dogs and cats has a wide range of presentations, ranging from general symptoms like fever and dyspnea to more specific signs involving neural, respiratory, cutaneous and ocular signs, thus a comprehensive differential diagnosis with leishmaniosis, neosporosis, and sarcocystosis constitute a key element. Because clinical disease is frequently associated with immunosuppressive therapy, an special interest should be paid to the serological status of *T. gondii* antibodies when corticosteroid drugs or transplant surgery are prescribed. In addition, there is a lack of knowledge regarding clinical consequences driven by venereal and congenital transmission. Finally, further studies aiming the isolation of *T. gondii* from clinical cases is of major interest to establish a possible association of strains causing specific clinical diseases and outcomes.

## Author Contributions

RC-B and SMG equally contributed with the acquisition and analysis of data, drafting, and critical revision of the manuscript.

### Conflict of Interest Statement

The authors declare that the research was conducted in the absence of any commercial or financial relationships that could be construed as a potential conflict of interest.
